# Isolated Central Nervous System Involvement after Brentuximab Vedotin Treatment for HIV-Positive ALK-Negative Anaplastic Large Cell Lymphoma

**DOI:** 10.1155/2024/5534556

**Published:** 2024-02-22

**Authors:** Takuya Suyama, Kumiko Matsui, Kosuke Makihara, Masatoshi Tsuru

**Affiliations:** ^1^Diabetes and Hematology Division, National Hospital Organization Kanmon Medical Center, 1-1, Sotouracho, Shimonoseki, Yamaguchi 752-8510, Japan; ^2^Surgical Pathology, Kyushu Rosai Hospital, 1-1 Sonekitamachi, Kokura Minami-Ku, Kitakyushu, Fukuoka 800-0296, Japan

## Abstract

Human immunodeficiency virus (HIV)-associated lymphoma poses a high mortality risk despite antiretroviral therapy (ART). Although intermediate- or high-grade B-cell lymphomas are common, anaplastic large-cell lymphomas (ALCLs) are rare and seldom affect the central nervous system (CNS). Herein, we present a case of HIV-associated ALCL with isolated CNS involvement that occurred following the discontinuation of ART that was administered after treatment with brentuximab vedotin (BV)—which does not cross the blood-brain barrier. At the time of CNS recurrence, the patient's CD4 count was 9 cells/mm^3^. This is the first report of CNS recurrence in HIV-associated ALCL. Considering the high risk of CNS relapse, we suggest initiating CNS prophylaxis in cases of HIV-associated ALCL, particularly in patients receiving CNS-impermeable agents such as BV.

## 1. Introduction

The incidence of human immunodeficiency virus (HIV)-associated lymphoma has declined in recent years, owing to the advent of antiretroviral therapy (ART); however, it continues to pose a high risk of mortality to the general population. Among the various subtypes, Burkitt's lymphoma and diffuse large B-cell lymphoma are the most prevalent, whereas anaplastic large cell lymphoma (ALCL) has a low incidence, accounting for <1% of all cases worldwide [1–3]. In patients with non-HIV-associated ALCL, central nervous system (CNS) involvement is rare. Brugières et al. reported that CNS relapse was observed in only 2 out of 352 patients with ALCL included in a randomized trial of an European Intergroup for Childhood Non-Hodgkin Lymphoma group [4]. No documented cases of CNS recurrence have been reported thus far in patients with HIV-associated ALCL. Herein, we present a unique case of HIV-associated ALCL with isolated CNS recurrence following ART discontinuation and treatment with brentuximab vedotin (BV) treatment. BV, which is used to treat both primary and recurrent ALCL, does not cross the blood-brain barrier (BBB) [5–7]. Moreover, HIV-associated lymphoma exhibits a tendency toward CNS involvement when the CD4+ cell count falls below 50/mm^3^ [8].

## 2. Case Presentation

A 46-year-old man presented to the hospital with oral ulcers, a mass on the right cheek, and fever. His medical history included adult-onset Still's disease that had been treated with prednisolone 2 years prior, and a *Pneumocystis pneumoniae* infection that had been treated with sulfamethoxazole-trimethoprim 1 year prior. Computed tomography (CT) revealed splenomegaly and multiple lymphadenopathies in the lungs. Positron emission tomography-CT (PET-CT) showed strong accumulation in sites corresponding to multifocal lymphadenopathies on both lung fields. Laboratory tests revealed a hemoglobin level of 9.9 g/dL and a C-reactive protein level of 2.2 mg/dL. In addition, the patient's soluble interleukin-2 receptor (sIL-2R) level had increased to 1,350 U/mL (reference range: 121–613 U/mL). His CD4+ cell count was 3 cells/mm^3^ and his HIV viral load was 45,000 copies/mL, leading us to make a diagnosis of HIV infection. A test for human T-cell leukemia virus type 1 (HTLV-1) was negative.

**Figure 1:**
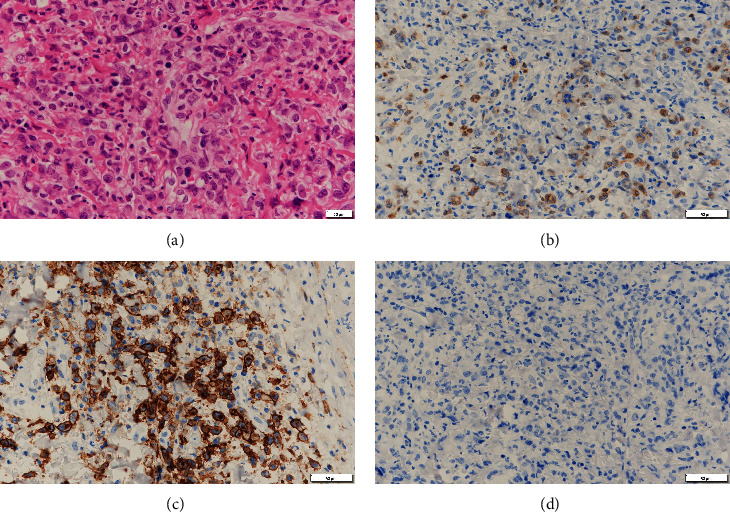
Diffuse infiltration of atypical lymphoid cells observed via hematoxylin and eosin (HE) staining (a) (bar = 20 *μ*m). Immunohistochemical staining of the atypical lymphoid cells was positive for CD3 (b), CD30 (c), and negative for anaplastic lymphoma kinase (ALK) (d) (bar = 50 *μ*m).

A biopsy of one of his oral ulcers revealed diffuse invasion of atypical lymphoid cells. Immunohistochemical staining of these cells was positive for CD3, CD30, and CD45 and negative for CD20 (Figure 1). Anaplastic lymphoma kinase (ALK) protein expression was negative, and the Ki67 index was 80% in the atypical cells. The patient was therefore diagnosed with ALK-negative anaplastic large-cell lymphoma. A CT-guided biopsy was performed on his pulmonary lymphadenopathies. This revealed infiltration of abnormal lymphoid cells positive for CD3 and CD30, resembling those of the oral ulcer lesion, which further confirmed ALCL infiltration. The patient was diagnosed with a stage IV, according to the Ann Arbor staging system. However, bone marrow aspiration and biopsy results did not indicate the presence of malignant lymphoma. The patient had an International Prognostic Index score of 2, indicating intermediate risk. He received cyclophosphamide, doxorubicin, vincristine, and prednisone (CHOP) chemotherapy. ART with lamivudine and raltegravir was initiated. Following six cycles of CHOP, a follow-up PET-CT confirmed the achievement of a first complete remission. However, 15 months after completing the CHOP regimen, another PET-CT revealed abnormal accumulation in the left abdominal lymph node, indicating relapse. The bone marrow examination revealed no other pathological lesions. His CD4+ cell count was 110 cells/mm^3^ at the time of the relapse. The patient then received BV therapy and achieved a second complete remission after 16 cycles (confirmed via another PET-CT scan). ART was voluntarily discontinued after 12 cycles of BV therapy.

**Figure 2:**
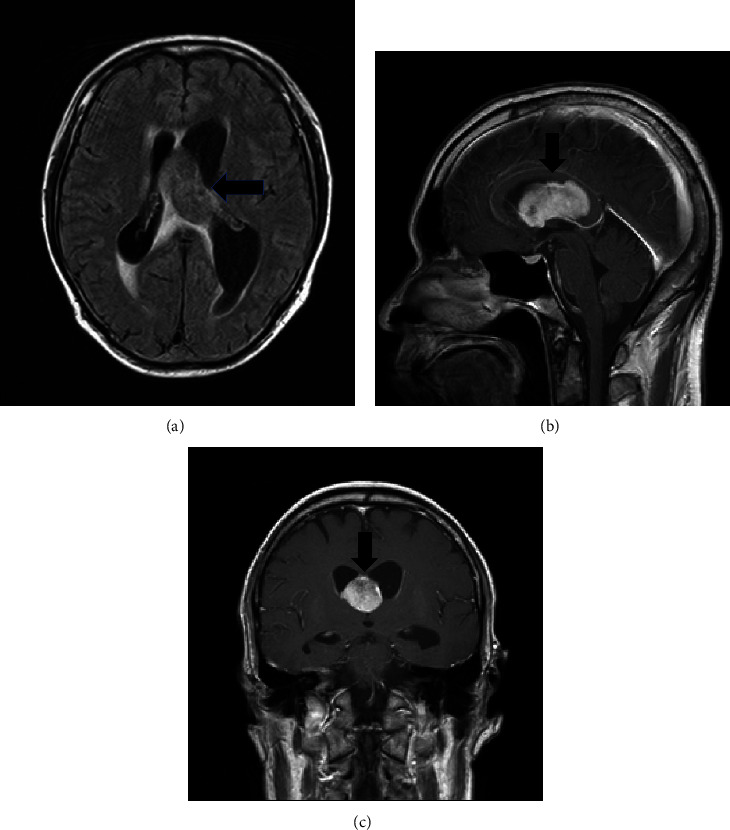
Magnetic resonance imaging (a)–(c) revealed a 4 cm lesion in the corpus callosum (indicated by the arrows). Panel (a) shows a fluid-attenuated inversion recovery image; (b) and (c) are T1-weighted images.

**Figure 3:**
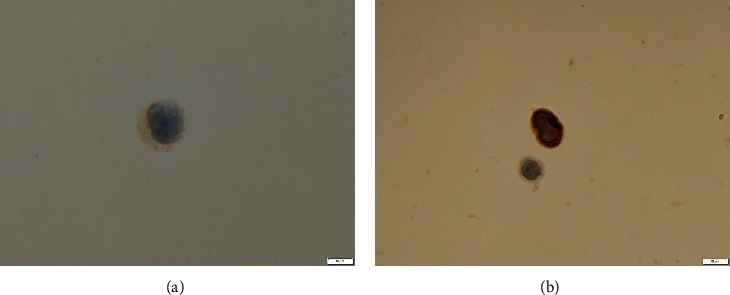
Irregularly-shaped lymphocytes in the patient's cerebrospinal fluid stained positive for CD3 (a) and CD 30 (b) (bar = 10 *μ*m).

Seven months after the final dose of BV was administered, the patient presented to the hospital once again—this time with functional decline and confusion. His CD4+ cell count was 294 cells/mm^3^ during ART, but subsequently dropped to only 9 cells/mm^3^ at the time of this second presentation to hospital. Brain magnetic resonance imaging (MRI) revealed a 4 cm lesion in the corpus callosum (Figure 2). Cerebrospinal fluid (CSF) analysis revealed an increase in cell count (21 cells/mm^3^) and protein concentration (10 mg/dL). The CSF cells exhibited irregular shapes and tested positive for CD3, CD30, and CD45 but negative for CD20 and CD79a (Figure 3). Whole-body CT and bone marrow examinations revealed no other pathological lesions. The patient's sIL-2R level was elevated, at 39,400 U/mL. He was therefore diagnosed with an isolated CNS relapse of ALCL.

High-dose systemic methotrexate was administered; however, the patient's symptoms continued to worsen. Subsequent brain MRI revealed an increase in the size of the multifocal-enhancing masses. The patient died 3 years and 6 months following his initial diagnosis.

## 3. Discussion

Patients infected with HIV are at high risk of developing malignant lymphomas, particularly intermediate- or high-grade B-cell lymphomas such as diffuse large B-cell lymphoma or Burkitt's lymphoma. The incidence of T-cell lymphoma in patients with HIV is reported to be ∼1–3%, with ALCL accounting for 22–28% [1, 2].

In recent years, the incidence of non-Hodgkin's lymphoma in patients with HIV has decreased, following the introduction of ART. However, patients with HIV still have a significantly higher risk of developing ALCL than the general population, particularly when their CD4 count falls below 200 cells/mm^3^ [2, 9].

Although T-cell ALCL is rare in patients with HIV, >100 cases of ALCL have been reported. A review of 86 cases and a case series of 37 patients revealed that the majority of these cases were ALK negative, and the incidence of ALCL increased significantly with a decrease in the CD4+ cell count. Approximately one-third of the patients tested positive for the Epstein-Barr virus, and 78% exhibited advanced lymphoma with extranodal involvement of organs such as the lungs, liver, bone marrow, spleen, and myocardium. The prognosis for this condition is poor, with a median survival of 5–6 months. ART has the potential to improve survival outcomes in HIV-associated ALCL [9, 10]. Moreover, other case reports support the finding that HIV-associated T-cell ALCL often advances with extranodal involvement and has a poor prognosis [2, 11].

An international clinical study of cases of pediatric non-HIV ALCL reported that CNS involvement at the time of diagnosis was rare, occurring in only 2.6% of the 463 patients analyzed [12]. In the ALCL99 study, among 352 patients without initial CNS involvement only two experienced CNS involvement at the time of first relapse [4]. The standard treatment for CNS involvement in ALCL remains undefined; however, multidrug chemotherapy with high-dose methotrexate, cytarabine, and intrathecal treatment is commonly administered—sometimes in conjunction with radiotherapy [6, 7, 12]. A review of 39 cases of primary CNS involvement in ALCL demonstrated that treatment with a high dose of methotrexate improved patient prognoses [13]. Only one case of HIV-associated ALCL with primary CNS involvement has previously been reported. That case involved a 46-year-old man who underwent whole-brain irradiation and subsequently died from *Aspergillus*-related pneumonia, 2 months following his diagnosis [14].

In the present case, the patient exhibited a low CD4+ count of 3 cells/mm^3^ at the time of diagnosis, suggesting that HIV infection may have contributed to the development of ALCL. He initially received CHOP therapy as the primary treatment, followed by BV as a salvage treatment following the first relapse. However, the patient experienced a relapse that involved the CNS, following the administration of BV.

Current evidence shows that BV does not cross the BBB [5–7]. Three cases of isolated CNS relapse without systemic recurrence have been reported following the administration of BV in the literature thus far [7, 15, 16]. These included the case of a 60-year-old woman with ALK-negative ALCL who experienced CNS relapse following eight cycles of BV during her third complete remission, a 19-year-old man with ALK-positive ALCL who experienced CNS relapse after four cycles of BV and crizotinib, and a 15-year-old boy with ALK-negative ALCL who relapsed following seven cycles of BV. No data exist in the literature regarding CNS prophylaxis in ALCL, and intrathecal methotrexate alone has been proven to be ineffective for preventing CNS relapse in patients with high-risk diffuse large B-cell lymphoma (DLBCL). By contrast, high-dose methotrexate has been reported to reduce CNS relapse [17, 18].

HIV-associated lymphoma frequently involves the CNS already, at the time of diagnosis. CNS involvement has been reported to be more prevalent when the CD4+ cell count is <50/mm^3^ [8]. In this case report, the patient discontinued ART, and his CD4+ count was only 9 cells/mm^3^ at the time of his CNS relapse. Considering that BV was administered as a single agent, this patient could have been considered to be at high risk of CNS relapse. Generally, CNS prophylaxis is not initiated during treatments for ALCL. However, in high-risk cases such as this one, CNS prophylaxis following relapse could have been considered. In the case of DLBCL, intrathecal methotrexate did not show any preventive effects against CNS relapse. Therefore, adding high-dose methotrexate as a CNS prophylaxis following relapse should be considered for patients with high-risk ALCL, even if it does not prove to be effective in this case [17, 18]. To the best of our knowledge, this is the first report of an isolated CNS relapse in HIV-associated ALCL. Our findings suggest that CNS prophylaxis should be considered for patients with HIV-associated ALCL—particularly in cases where ART is unsuccessful and when patients are receiving drugs such as BV that do not cross the BBB.

## Data Availability

The data can be made available from the corresponding author upon reasonable request.
